# A Carbon Nanotube Transistor Based on Buried-Gate Structure

**DOI:** 10.3390/ma18020218

**Published:** 2025-01-07

**Authors:** Haiou Li, Yuanmei Liao, Fabi Zhang, Tangyou Sun, Xingpeng Liu, Shouwei Chen

**Affiliations:** 1 Guangxi Key Laboratory of Precision Navigation Technology and Application, Guilin University of Electronic Technology, Guilin 541004, China; lihaiou@guet.edu.cn (H.L.); liao_ymei@163.com (Y.L.); zhangfabi@outlook.com (F.Z.); suntangyou@guet.edu.cn (T.S.); 2 School of Microelectronics and Artificial Intelligence, Kaili University, Kaili 556011, China; 3 Micronano and Intelligent Manufacturing Engineering Research Centre of Ministry of Education, Kaili 556011, China

**Keywords:** carbon nanotube transistors, thin-film transistor, surface planarization

## Abstract

From the discovery of carbon nanotubes to the ability to prepare high-purity semiconductor carbon nanotubes in large quantities, the large-scale fabrication of carbon nanotube transistors (CNT) will become possible. In this paper, a carbon nanotube transistor featuring a buried-gate structure, employing an etching process to optimize the surface flatness of the device and enhance its performance, is presented. This CNT thin-film transistor has a current switching ratio of 10^4^, a threshold voltage of around 1 V, and a mobility that can reach 6.95 cm^2^/V·s, indicating excellent electrical performance. The device achieves operational targets at low voltage, facilitating the development of small and portable electronic devices.

## 1. Introduction

For over half a century, the advancement of silicon-based integrated circuits has precipitated substantial transformations in the field. However, with the advent of the post-Moore era, the continuous scaling of technology nodes has encountered significant challenges, including increased power consumption, escalating design costs, and heightened process complexity. These factors have pushed silicon technology to the limits of physical and engineering feasibility, underscoring the necessity for alternative semiconductor materials [1,2]. In comparison with conventional silicon processing, carbon-based methods can potentially halve the fabrication flow. Although current mainstream technologies lag three generations behind silicon processes, carbon-based chips can achieve comparable performance and integration levels [3,4,5].

Carbon nanotubes (CNTs), as a distinctive class of carbon-based materials, exhibit exceptional structural characteristics alongside superior mechanical and electrical properties [6,7]. CNTs can be categorized finely, with single-walled carbon nanotubes (SWCNTs) further classified based on chirality [8]. Approximately one-third of these display metallic behavior, qualifying them as conductors, while the remaining two-thirds exhibit semiconducting properties, functioning effectively as channel materials. SWCNTs possess diameters around 1 nm and an intrinsic mobility of 1 × 10^5^ cm^2^/V·s [9,10]. Moreover, CNTs exhibit high solution processability, enabling the fabrication of devices at low temperatures and cost-efficiency [11,12,13]. However, current device structures and fabrication techniques face several challenges, including non-uniform deposition of gate dielectrics, increased interfacial scattering, and degraded device performance.

Based on the position of the gate, the device structure can be classified into three types: top-gate, back-gate, and dual-gate structures. In the top-gate fabrication process, carbon nanotubes need to be deposited onto the substrate first. However, during the atomic layer deposition (ALD) process, the absence of dangling bonds on the surfaces of carbon nanotubes leads to non-uniform deposition of the gate dielectric on the channel, making scaling very difficult [14]. Many studies have repeatedly attempted to apply thin gate dielectrics to carbon-based surfaces, using methods including metal or nanoparticle coatings, along with the application of molecules for surface modification or functionalization [15,16]. However, various approaches often result in degraded dielectric quality and increased interfacial scattering, leading to reduced electrical performance of the FETs. Both structures provide maximum protection to the channel material. The advantage of the buried-gate structure lies in enabling single control of the device. The dual-gate structure is suitable for high-frequency devices [17,18,19]. Recent advancements in materials, such as metamaterials and graphene, have opened up new avenues for device design and applications [20,21]. However, the lack of an intrinsic bandgap in graphene and the complexity of fabricating metamaterial-based devices currently limit their direct application in transistor designs. In contrast, CNT-based devices offer tunable bandgaps, high mechanical flexibility, and compatibility with low-temperature processes, making them particularly suited for the buried-gate architecture explored in this study.

Current research on carbon nanotube (CNT) transistors focuses on improving the density and uniformity of thin films, with the device structure typically being full back-gate [22,23,24]. In contrast, the present work introduces a buried-gate structure and investigates and analyzes CNT transistors with this configuration. Unlike top-gate devices, this approach eliminates issues related to the deposition of gate dielectrics while maintaining the advantages of precise gate control. The proposed structure leverages a simplified process flow, achieving low-temperature, low-cost manufacturing while maintaining high performance.

## 2. Experiment

**Figure 1:**
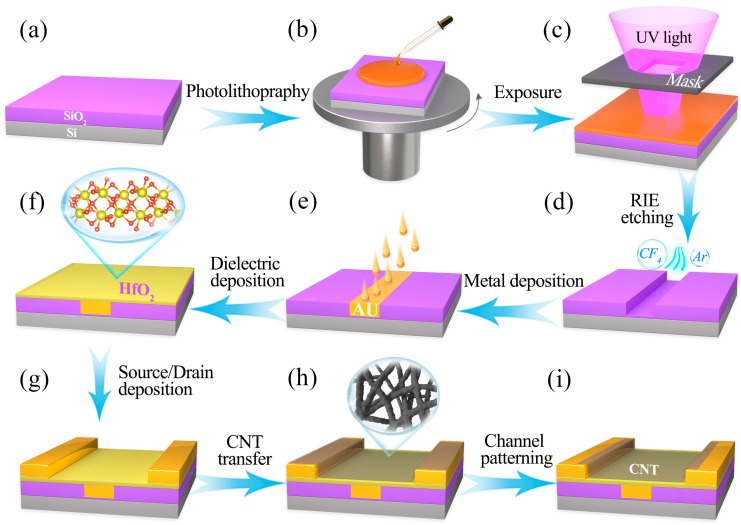
Schematic diagram of the fabrication process for CNT with a buried-gate structure. (**a**) Transistor substrate; (**b**) Photoresist coating; (**c**) UV exposure; (**d**) RIE etching; (**e**) Gate electrode deposition; (**f**) Gate dielectric deposition; (**g**) Source/drain metal deposition; (**h**) CNT deposition; (**i**) Channel patterning.

The schematic in Figure 1 illustrates the primary processes involved in fabricating CNT with buried-gate structures. All experiments in this study were conducted in a cleanroom environment at room temperature. The initial step should prepare a pattern on the substrate using photolithography. To facilitate the stripping process and minimize metallic residues, the first layer of photoresist used was the non-photosensitive LOR-3A. The photolithography chemicals used in this experiment were provided by Xi’an Boyan Micro-Nano Information Technology Co., Ltd., Xi’an, China. This photoresist is typically composed of high-molecular-weight polymeric materials, such as polymethyl methacrylate (PMMA) or polyimide, resulting in a viscosity that is greater than conventional photoresists. To achieve uniform coverage of the surface, the prespin speed had to be higher than that for standard photoresists; thus, a rotational speed of 3000 r/min was employed for 60 s, followed by a baking step on a preheated hot plate at 175 °C for 300 s. The second layer consisted of positive photoresist S1813, which was applied at a rotational speed of 4000 r/min for 60 s, followed by baking at 115 °C for 60 s (Figure 1b). A 9 s ultraviolet (UV) exposure was subsequently conducted (Figure 1c). The UV photolithography machine used in this study was model H94-25. Reactive ion etching (RIE) was then employed to etch the silica and form the gate recesses (Figure 1d). The RIE system used in this study was provided by Japan Electron Optics Laboratory, Tokyo, Japan. The machine’s RF power was set to 100 W, with CF_4_ used as the etching gas at a flow rate of 70 sccm, while Ar served primarily as the carrier gas at a flow rate of 30 sccm. Individual electrodes were created by depositing a 50 nm thick metallic layer via a thermal evaporation deposition system (Figure 1e).

**Figure 2:**
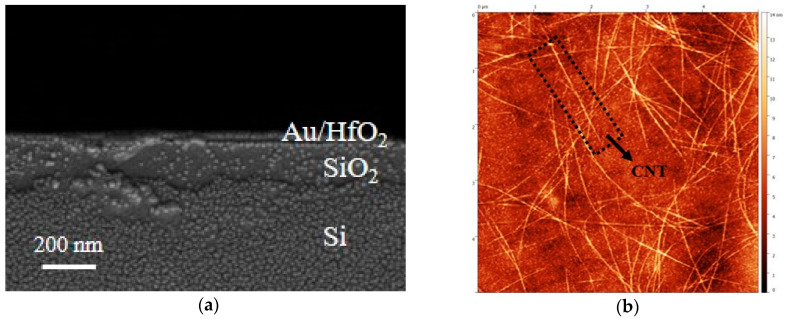
(**a**) SEM image of the CNT; (**b**) AFM image of CNT deposition.

Next, a 20 nm thick layer of HfO_2_ was grown by ALD at 92 °C, establishing the dielectric layer (Figure 1f). The ALD system used in this study is model R-200. HfO_2_, as a high-κ dielectric layer, can induce a stronger electric field effect at lower gate voltages. Due to its high dielectric constant, HfO_2_ allows for the same electric field strength with a thicker gate dielectric, thereby reducing gate leakage current and effectively improving the static power consumption performance of the device. Following this, a 100 nm layer of source–drain metal was deposited through thermal evaporation, maintaining a spacing of 5 µm, and excess metal was removed using an NMP solution (Figure 1g). The CNT dispersion employed in this study was sourced from Suzhou Semiconductor Company. The CNTs underwent a 24 h soaking period at room temperature, followed by rinsing with xylene to eliminate excess material, and isopropanol was used to remove any residual solvents (Figure 1h). To enhance the adhesion of the CNTs to the substrate, a baking process at 120 °C for 15 min was performed. To address channel adhesion issues, oxygen plasma etching was conducted to create a disjointed pattern, utilizing a flow rate and power set at 50 sccm and 50 W and an etching duration of 30 s (Figure 1i). The dimensions, geometries, and layouts of the channels were meticulously controlled to optimize device performance. Figure 2a shows the side view SEM image of the CNT FET fabricated on a Si/SiO_2_ substrate. The image qualitatively illustrates the layered structure of the device, including the Si substrate, the SiO_2_ layer, and the deposited Au/HfO_2_ layer. This SEM observation provides insights into the uniformity and morphology of the layers, which are critical for understanding the fabrication process. However, it is noted that SEM has limitations in providing detailed cross-sectional structural analysis. Advanced characterization methods, such as TEM, are suggested for future studies to achieve a more comprehensive evaluation of the device’s sidewall structure. While Figure 2b displays the AFM image illustrating the CNT deposition. Its average surface roughness is 1.07 nm. It can be seen that the length of the carbon nanotubes reaches the micron scale. Longer carbon nanotubes can reduce contact resistance and scattering effects, thereby improving carrier mobility. The micron-scale length allows electrons to have a longer free transport distance, reducing resistance caused by grain boundaries or defects and enhancing the overall current transport efficiency.

## 3. Results and Analysis

**Figure 3:**
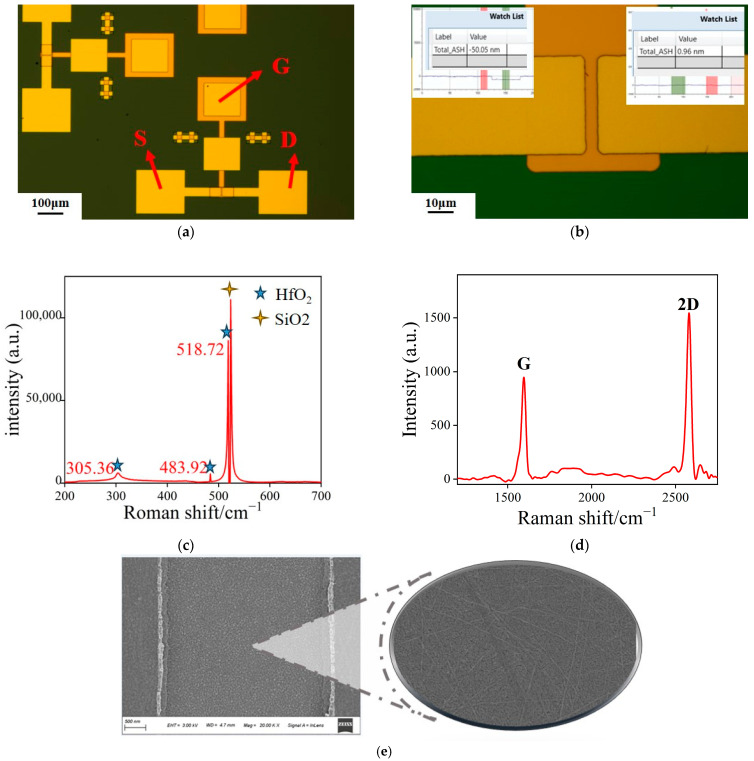
(**a**) Optical micrograph of the CNT FET; (**b**) optical microscope image of the CNT FET channel; (**c**) Raman spectrum of the gate dielectric HfO_2_; (**d**) Raman spectrum of the conductive channel CNT; (**e**) SEM image of the conductive channel.

Initially, Raman spectroscopy and SEM were employed to characterize the device and confirm the presence of HfO_2_ and CNTs in the sample. The optical microscopy image of the CNT FET is presented in Figure 3a, while the magnified view in Figure 3b reveals that the length of the conducting channel is 5 μm, with source and drain electrode lengths of 40 μm and a gate electrode length of 70 μm. The image on the left shows the results of measurements taken using the step profiler. The groove, which appears after RIE etching, has a depth of 50.05 nm. The image on the right shows the measurement of the deposited gate metal using the step profiler, with a surface error of only 0.96 nm. The gate dielectric was characterized using Raman spectroscopy, with Figure 3c displaying the characteristic peaks of HfO_2_. The Raman spectrum of CNTs features two critical peaks: the 2D peak (G’ peak) and the G peak. The relative intensities of these peaks can vary due to several factors, including the type, quality, chirality, and diameter of the sample, as well as the wavelength of the laser used. For single-walled CNTs, the G peak intensity is generally weaker than that of the 2D peak due to the electronic structure of single-walled CNTs resembling that of a one-dimensional graphene sheet, mirroring the Raman characteristics of graphene. In contrast, multi-walled CNTs typically exhibit a higher G peak intensity compared with the 2D peak, attributed to their more complex structure and the interactions between multiple layers, which modify their electronic band structure and result in a relatively weaker 2D peak intensity. The design of the buried-gate structure minimizes the impact of process steps or experimental reagents on the CNT material during the experiment. Figure 3d reveals an absence of the D peak around 1350, with the G peak and 2D peak clearly visible. The intensity ratio (I_2D_/I_G_) of the G peak to the 2D peak is 1.62, confirming the high quality of the carbon nanotubes and that they are predominantly single-walled. Figure 3e provides a SEM image of the CNT channel.

**Figure 4:**
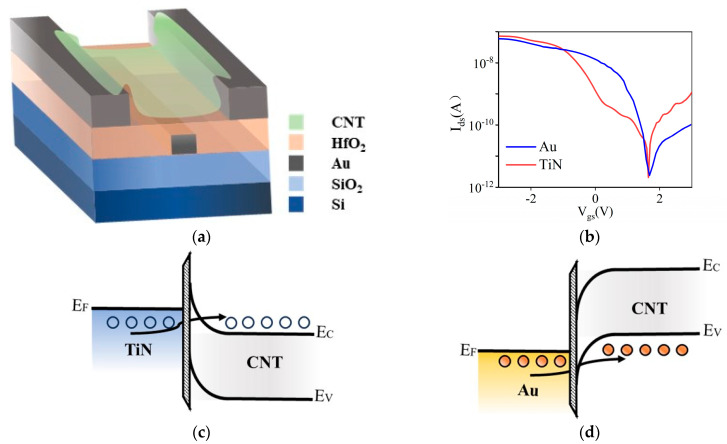
(**a**) Schematic diagram of the buried-gate transistor without RIE etching; (**b**) comparison of transfer characteristics for TiN and Au electrode transistors; (**c**) energy band diagram of the TiN-CNT contact; (**d**) energy band diagram of the Au-CNT contact.

The electrical characteristics of the device were measured using a Keithley 2636B semiconductor parameter analyzer, which offers a current resolution of 0.1 fA and a voltage resolution of 0.1 mV. All measurements were performed at room temperature under ambient conditions to ensure reliable and reproducible results. The devices with two different metal electrodes were compared first. Figure 4a depicts the cross-section of the back-gated CNT FET without RIE etching. Au and TiN were used to compare the effects of these two different conductive materials on the transistor’s performance. Figure 4b illustrates the transfer characteristics of the two transistors, both of which display transfer curves typical of P-type CNT FETs. Although the Dirac points of the two devices—defined as the gate voltage at which the source–drain current is minimized—were relatively close at approximately 1.75 V, the transistor with the TiN electrode exhibited typical bipolar behavior. Based on the energy band diagram analysis, the work functions of carbon nanotubes and TiN are typically 4.8 eV and 4.6 eV, respectively. This is illustrated in Figure 4c: when the positive voltage is applied to the CNT, the energy bands bend downward, reducing the barrier thickness between the source electrode and the CNT. This transport mechanism is electron conduction, increasing the likelihood of electron tunneling from the electrode into the CNTs, thus displaying n-type transport characteristics. Conversely, the work function of Au is 5.1 eV (as shown in Figure 4d). When a negative voltage is applied, holes can be injected into the channel from the electrode through a low barrier. And the potential barrier thickens under positive voltage, effectively suppressing the bipolar behavior associated with the Au electrode.

**Figure 5:**
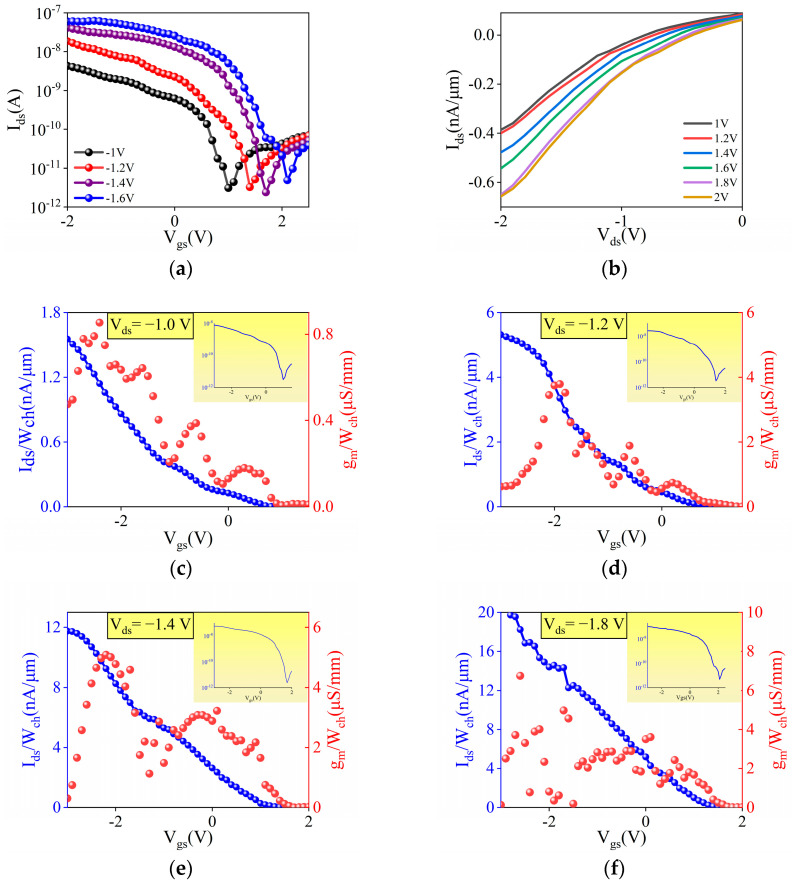
(**a**) Transfer characteristics curve of the transistor without RIE etching; (**b**) output characteristics curve of the transistor without RIE etching; transconductance (gm) of the transistor without RIE etching at (**c**) V_ds_ = −1.0 V, (**d**) V_ds_ = −1.2 V, (**e**) V_ds_ = −1.4 V, and (**f**) V_ds_ = −1.8 V.

Figure 5a presents the transfer characteristics and output characteristic curves of the gold electrode CNT device, achieving an I_on_/I_off_ ratio of up to 10^3^. The I_on_/I_off_ ratio is a key metric for digital logic applications, calculated as the ratio of the maximum current to the minimum current observed within the V_gs_ scanning window. The figure illustrates a gradual shift of the Dirac point toward positive voltages. This shift occurs because, as V_ds_ increases, the degree of energy band bending also increases, facilitating the movement of holes between the source and drain, thus strengthening the influence of the electric field. This is a normal phenomenon. In circuit design, techniques such as negative feedback are commonly employed to mitigate offset errors. The output characteristics are shown in Figure 5b. As the channel length of the device decreases, particularly in the micro-nanometer scale, the gate’s control over the charge carriers weakens. This reduction leads to increased leakage current, which becomes particularly pronounced even at low gate voltages (V_ds_ = 0). The increase in V_gs_ enhances the concentration of carriers within the channel, thereby increasing conductivity. Consequently, even in the absence of an applied voltage, holes can diffuse due to the concentration gradient.

Moreover, the surface flatness of the device significantly impacts its interfacial states. Suboptimal interfacial conditions can lead to a higher density of surface states that readily capture carriers, contributing to an elevated threshold voltage. Currently, numerous techniques for optimizing surface flatness have emerged, highlighting the growing importance of this aspect. For instance, in the field of semiconductor manufacturing, various methods of Chemical Mechanical Polishing (CMP) have been developed, alongside extensive research on advanced thin-film deposition processes. As illustrated in Figure 5c–f, the threshold voltage (Vth) is approximately −2 V, established as the gate voltage at which the transconductance (g_m_) reaches its maximum value. In Figure 5c (V_ds_ = −1.2), the g_m_ exhibits a moderate peak, reflecting limited carrier mobility under a lower electric field. With the increase in V_ds_ to −1.8 V in Figure 5f, the peak g_m_ becomes significantly higher. This increase can be attributed to the enhanced electric field across the channel, which accelerates carrier drift velocity and thus improves channel conductivity. The trend demonstrates that a higher V_ds_ effectively facilitates charge transport within the device. The I_on_ steadily increases as V_ds_ rises. This trend is attributed to the combined effects of channel length modulation and enhanced conductivity, which together contribute to a higher switching ratio. As V_ds_ increases from Figure 5c to Figure 5f, these effects become more pronounced, resulting in a larger separation between the I_on_ and I_off_, thereby improving the device’s overall switching performance. To enhance device performance, it is imperative to address surface defects and impurities to reduce the interface state density, thereby improving the overall electrical characteristics of the device. As semiconductor technology nodes continue to advance, device scaling will impose stricter requirements on surface flatness to prevent dimensional variations. Fluctuations in surface flatness can result in uneven physical thicknesses in the gate and source–drain regions. Variations in gate dielectric thickness directly impact electrical performance, such as Vth shifts. Superior flatness will better protect the channel materials in buried-gate devices, thereby ensuring high yield. In future large-scale manufacturing, it will help prevent local failures caused by variations across different wafer regions.

**Figure 6:**
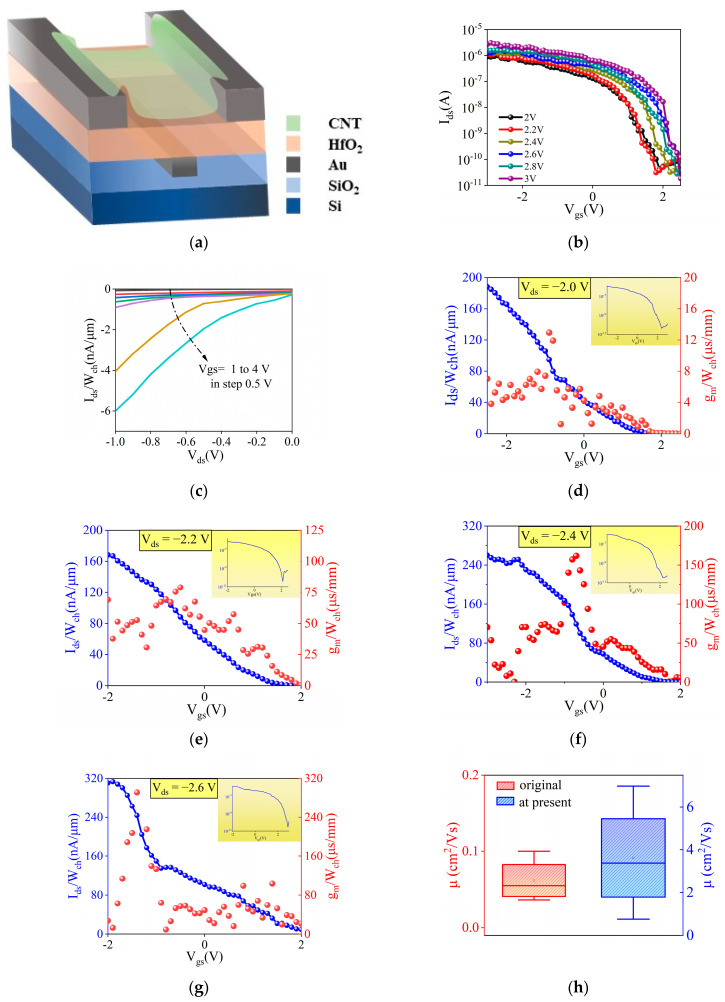
(**a**) Schematic diagram of the buried-gate structure transistor; (**b**) transfer characteristics curve of the buried-gate structure transistor; (**c**) output characteristics curve of the buried-gate structure transistor; transconductance (gm) of the buried-gate structure transistor at (**d**) V_ds_ = −2.0 V, (**e**) V_ds_ = −2.2 V, (**f**) V_ds_ = −2.4 V, and (**g**) V_ds_ = −2.6 V; (**h**) statistical graph of transistor mobility before and after etching treatment.

In order to improve the surface flatness and enhance the electric field uniformity, the buried gate prepared in this paper was first plasma etched to create a notch structure, and then the appropriate thickness of metal was deposited (as shown in Figure 6a), with an etching depth of 50 nm. This process is very critical and is an important step to improve the interface state. To more effectively assess the performance of the CNT TFT following the enhancement, the device performance was analyzed under a typical transistor operating voltage of 2–3 V. The switching ratio improved by an order of magnitude to more than 10^4^ (Figure 6b), and the Dirac point positive bias trend of the Dirac point was reduced compared with the unetched transistor. This indicates that the variation in energy band curvature is reduced during operation, resulting in improved transistor stability. As seen in the output characteristic curve (Figure 6c), when V_ds_ is 0, the leakage current decreases. The etching of the gate notch reduces the effect on the flatness of the gate dielectric film and improves the interface between the CNT and the insulating layer. At a gate voltage of approximately 1 V, the device achieved transconductance (as shown in Figure 6d–g). This indicated that the threshold voltage of the device was reduced by half. Figure 6d–g present the electrical characteristics of the optimized device under increasing V_ds_.

Even though the Vth decreases, the switching ratio and g_m_ remain unaffected due to the improved flatness of the device surface. The increase in V_ds_ from −2.0 V to −2.6 V demonstrates enhanced current driving capability. This behavior is typical, as a higher V_ds_ facilitates greater carrier transport through the device channel. Specifically, the transconductance values are approximately 14 μS/mm in Figure 6d, 77 μS/m in Figure 6e, 170 μS/mm in Figure 6f, and 300 μS/m in Figure 6g. The progressive increase in the peak transconductance values with V_ds_ indicates improved gain performance under higher drain voltages. Furthermore, the slight shift in the peak positions with varying V_ds_ suggests a change in the device’s optimal operating point. These characteristics collectively demonstrate the ability of the optimized device to maintain robust performance under different V_ds_ conditions. At typical operating voltages, the increase in the switching ratio, turn-on voltage, and transconductance with higher V_ds_ highlights the device’s capability to operate more efficiently under elevated electric field conditions.

Across all V_ds_ conditions, the device maintains steep and stable subthreshold slopes, while the increasing separation between I_on_ and I_off_ results in a higher switching ratio. These results illustrate the consistent and robust performance of the same device under varying electric fields, highlighting its suitability for high-performance applications. This results in enhanced drive capability for IC applications, enabling higher I_ds_ at lower gate voltages. However, reducing Vth also introduces challenges associated with short-channel effects, such as drain-induced barrier lowering (DIBL), which weakens the gate control over the channel. This is particularly significant for low-power devices designed at scaled technology nodes, where achieving an optimal Vth is critical to ensuring device stability and efficiency. Notably, with the optimized device, its switching characteristics do not degrade despite the reduced Vth. In Figure 6g, the transconductance g_m_ is approximately 1.46 µS, which is considered moderate for high-mobility materials. While higher transconductance is typically associated with increased current drive capability and higher power consumption, in low-power applications, it allows the device to operate efficiently at lower V_gs_. Consequently, this device is well suited for low-power applications, particularly in high-mobility materials or optimized devices, where it can sustain low power consumption while providing superior switching performance. The measured transconductance g_m_ demonstrates that the device maintains sufficient signal amplification capability within the low-power operational range, indicating potential for low-noise and energy-efficient amplification circuits. Furthermore, the mobility is significantly enhanced, as shown in Figure 6h. The experimental findings demonstrate the excellent low-power characteristics of the device, with a power consumption range of 1–30 nW under varying V_gs_ and V_ds_ conditions (Figure S1). These results suggest strong potential for applications in energy-sensitive fields, such as IoT sensor nodes, wearable electronics, and self-powered systems. The ability to effectively control power dissipation by tuning V_gs_ provides valuable insights for optimizing transistor designs tailored to ultra-low-power applications. Furthermore, the favorable transconductance g_m_ and I_on_/I_off_ ratio observed in this study indicate that the device can support efficient signal amplification and low-leakage switching, making it a promising candidate for both analog and digital low-power circuits.

The subthreshold slope (SS) of the device is approximately 303.07 mV/dec. Although higher than the theoretical limit of 60 mV/dec, this value is acceptable for carbon-based devices in practical experiments. However, it reflects the current limitations in material quality and interface engineering during the device fabrication process. Notably, the subthreshold slope remains consistent under different voltage conditions, indicating stable subthreshold characteristics within the specified region. In the low-voltage range of 0.05 V to 0.2 V, the on-resistance (R_on_) of the device is calculated to be approximately 20 MΩ. For experimental devices, an R_on_ in the range of 10–30 MΩ is reasonable, particularly in cases where the carbon nanotube density is low or the contact resistance is high. Future research will focus on optimizing the alignment and increasing the density of carbon nanotube arrays to enhance the activity of the conduction channel, thereby improving subthreshold performance and reducing R_on_.

Table 1 compares the performance of CNT-TFTs across different studies, highlighting key metrics such as width-to-length ratio (W/L), switching ratio (I_on_/I_off_), and mobility (µ). In our work, the buried-gate structure achieves a competitive switching ratio of 10^4^, comparable to the highest-performing studies, while demonstrating a mobility of 6.95 cm^2^/V·s, which is significantly higher than most cited works. The large W/L ratio (100/5 µm) in our devices ensures higher current output, though further optimization of CNT alignment and density could potentially enhance mobility. Compared with studies with lower I_on_/I_off_ or µ, our results suggest that the buried-gate structure improves interface uniformity and reduces scattering. However, further efforts to balance mobility and power efficiency, as well as long-term stability tests, will help solidify the advantages of this architecture for practical.

## 4. Conclusions

In this paper, we introduced a CNT FET based on a buried-gate structure. We analyzed the impact of conductive materials with different work functions, TiN and Au, on transistor polarity through comparison and optimization of the gate structure. Our study demonstrated that the uniformity of the device surface significantly influences the performance of the CNT FET. After etching the substrate, the integration of the buried-gate structure enhances the device characteristics by reducing the density of interface states, thereby improving the uniformity of the electric field distribution. As a result, the switching ratio increased by an order of magnitude, and the Vth was reduced from 2 V to around 1 V compared with conventional devices. Furthermore, the response speed of the device was improved, enabling operation at lower voltages, which is conducive to the development of small and portable electronic devices. However, it is important to note that the device may be subject to certain physical and chemical limitations during practical use. Environmental factors such as humidity and high temperatures could potentially affect the stability of the device and the performance of its sensors. To ensure the reliability of the device in harsh conditions, further optimization of its design may be necessary. While the buried-gate structure offers a simplified fabrication process and provides maximum protection for the channel material, it also exposes carbon nanotubes directly to the ambient environment. Therefore, it is crucial to explore effective passivation layers. These passivation layers should prevent the channel material from interacting with air and moisture, thereby enhancing device reliability and prolonging its operational lifetime. To overcome these challenges, further advancements in novel materials, such as high-κ dielectric layers, and innovations in fabrication techniques, such as 3D structural designs, will likely be required in the future.

## Figures and Tables

**Table 1:** Comparison of the performance of CNT-TFTs between the cited literature and this work.

Reference	W/L (μm)	I_on_/I_off_	μ (cm/V·s)
[23]	4/0.15	10^3^	0.14
[25]	50/NA	10^5^	0.5
[26]	365/80	10^3^	0.015
[27]	1250/85	10^4^	4.3
[28]	NA	10^4^	1
[29]	25/3	10^3^	0.01
This work	100/5	10^4^	6.95

## Data Availability

The data presented in this study are available upon request from the corresponding author. The data are not publicly available due to privacy considerations.

## Supplementary Materials

The following supporting information can be downloaded at https://www.mdpi.com/article/10.3390/ma18020218/s1, Figure S1: Power consumption characteristics of the optimized transistor; Figure S2: SEM image of soaking CNT solution for 12 h/16 h/24 h/28 h.

## References

[1] Guisinger N.P., Arnold M.S. (2010). Beyond silicon: Carbon-based nanotechnology. MRS Bull..

[2] Zhang X., Zhu H., Xiong G., Zhu C., Huang X., Cao S., Zhang J., Yan Y., Yao Y., Zhang D. (2021). Radiation-hardened property of single-walled carbon nanotube film-based field-effect transistors under low-energy proton irradiation. J. Semicond..

[3] Wang C., Takei K., Takahashi T., Javey A. (2013). Carbon nanotube electronics–moving forward. Chem. Soc. Rev..

[4] Cao Q. (2021). Carbon nanotube transistor technology for More-Moore scaling. Nano Res..

[5] Hu Y., Peng L.-M., Xiang L., Zhang H. (2020). Flexible integrated circuits based on carbon nanotubes. AMR.

[6] Bernholc J., Brenner D., Buongiorno Nardelli M., Meunier V., Roland C. (2002). Mechanical and electrical properties of nanotubes. Annu. Rev. Mater. Sci..

[7] Zhu Q.-B., Li B., Yang D.-D., Liu C., Feng S., Chen M.-L., Sun Y., Tian Y.-N., Su X., Wang X.-M. (2021). A flexible ultrasensitive optoelectronic sensor array for neuromorphic vision systems. Nat. Commun..

[8] Abd I.K., Shano A.M., Khodair Z.T. (2022). MWCNT Thin Films by CVD Method and Some Applications. J. Nano-Electron. Phys..

[9] Henni A., Harfouche N., Karar A., Zerrouki D. (2021). Solar cell based on carbon and graphene nanomaterials. Sustainable Material Solutions for Solar Energy Technologies.

[10] Fagan J.A., Hároz E.H., Ihly R., Gui H., Blackburn J.L., Simpson J.R., Lam S., Hight Walker A.R., Doorn S.K., Zheng M. (2015). Isolation of >1 nm diameter single-wall carbon nanotube species using aqueous two-phase extraction. ACS Nano.

[11] Purohit R., Purohit K., Rana S., Rana R., Patel V. (2014). Carbon nanotubes and their growth methods. PMS.

[12] Qiu S., Wu K., Gao B., Li L., Jin H., Li Q. (2019). Solution-processing of high-purity semiconducting single-walled carbon nanotubes for electronics devices. Adv. Mater..

[13] Liu L., Han J., Xu L., Zhou J., Zhao C., Ding S., Shi H., Xiao M., Ding L., Ma Z. (2020). Aligned, high-density semiconducting carbon nanotube arrays for high-performance electronics. Science.

[14] Xuan Y., Wu Y., Shen T., Qi M., Capano M.A., Cooper J.A., Ye P. (2008). Atomic-layer-deposited nanostructures for graphene-based nanoelectronics. Appl. Phys. Lett..

[15] Jung H., Park J., Oh I.-K., Choi T., Lee S., Hong J., Lee T., Kim S.-H., Kim H. (2014). Interfaces, Fabrication of transferable Al_2_O_3_ nanosheet by atomic layer deposition for graphene FET. ACS Appl. Mater. Interfaces.

[16] Lee S.K., Kim Y.J., Heo S., Park W., Yoo T.J., Cho C., Hwang H.J., Lee B. (2019). Technology, Advantages of a buried-gate structure for graphene field-effect transistor. Semicond. Sci. Technol..

[17] Wang F., Xiaoli T., Jiang L., Yun B. (2020). Development Overview of Silicon Carbide Insulated Gate Bipolar Transistor. Power Electron..

[18] Sarkar A., Das A.K., De S., Sarkar C. (2012). Effect of gate engineering in double-gate MOSFETs for analog/RF applications. Microelectron. J..

[19] Rashid S., Bashir F., Khanday F.A., Beigh M.R., Hussin F. (2021). 2-D design of double gate Schottky tunnel MOSFET for high-performance use in analog/RF applications. IEEE Access.

[20] Mohammadi Estakhri N., Edwards B., Engheta N. (2019). Inverse-designed metastructures that solve equations. Science.

[21] Akbari M., Shahbazzadeh M.J., La Spada L., Khajehzadeh A. (2021). The graphene field effect transistor modeling based on an optimized ambipolar virtual source model for DNA detection. Appl. Sci..

[22] Xun Y. (2020). Study on Experimental Deposition of Carbon Nanotube Film on Wedge Wafer. Res. Des..

[23] Yang L., Li H., Xiu H., Deng M., Tian Q., Zhang Q., Xin X. (2023). Scaling carbon nanotube field effect transistors to 30 nm channel length on pretreated PET. Carbon.

[24] Ye H., Wang H., Yu X., Yao Y., Xiang L. (2023). Evaluation of influence of substrate planarization on the uniformity of flexible carbon nanotube thin film transistors. IEEE Trans. Electron. Devices.

[25] Li H., Liu H., Tang Y., Guo W., Zhou L., Smolinski N. (2016). Interfaces, Electronically pure single-chirality semiconducting single-walled carbon nanotube for large-scale electronic devices. ACS Appl. Mater. Interfaces.

[26] Lee W., Koo H., Sun J., Noh J., Kwon K.-S., Yeom C., Choi Y., Chen K., Javey A., Cho G. (2015). A fully roll-to-roll gravure-printed carbon nanotube-based active matrix for multi-touch sensors. Sci. Rep..

[27] Lau P.H., Takei K., Wang C., Ju Y., Kim J., Yu Z., Takahashi T., Cho G., Javey A. (2013). Fully printed, high performance carbon nanotube thin-film transistors on flexible substrates. Nano Lett..

[28] Wang H., Cobb B., van Breemen A., Gelinck G., Bao Z. (2014). Highly stable carbon nanotube top-gate transistors with tunable threshold voltage. Adv. Mater..

[29] Kimbrough J., Williams L., Yuan Q., Xiao Z. (2020). Dielectrophoresis-based positioning of carbon nanotubes for wafer-scale fabrication of carbon nanotube devices. Micromachines.

