# Supplementary material for: A Carbon Nanotube Transistor Based on Buried-Gate Structure
Source: Materials (Basel). 2025 Jan 7;18(2):218. doi: 10.3390/ma18020218 (PMC11767110; doi:10.3390/ma18020218)
Supplement: Supplementary file 1 [file materials-18-00218-s001.zip › materials-3358946-supplementary.pdf]

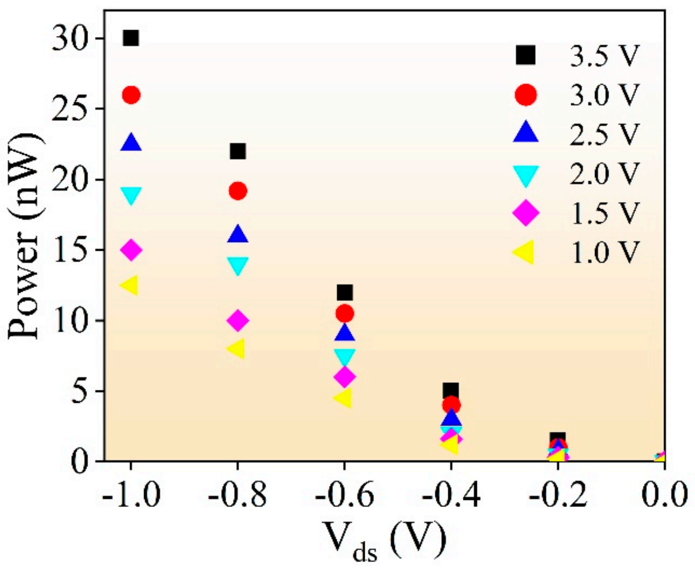

Figure S1. Power consumption characteristics of the optimized transistor.

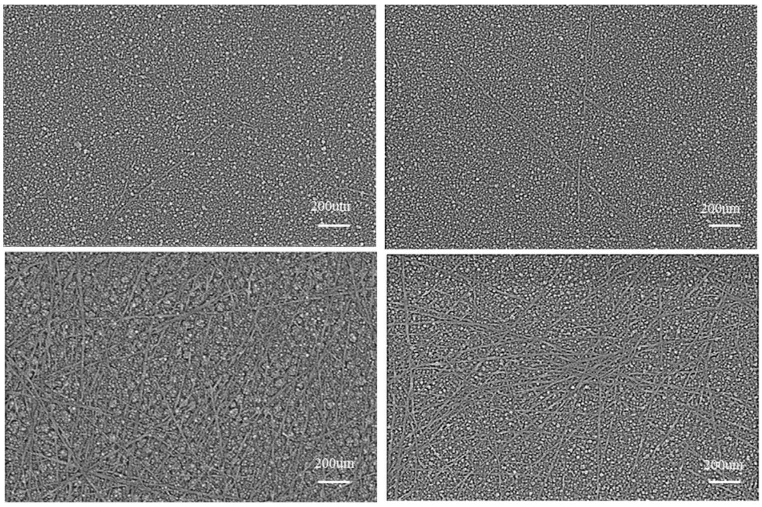

Figure S2. SEM image of soaking CNT solution for 12 h/16 h/24 h/28 h.
